# Patient-Based Dose Audit for Common Radiographic Examinations With Digital Radiology Systems: A Retrospective Cross-Sectional Study

**DOI:** 10.7759/cureus.15005

**Published:** 2021-05-13

**Authors:** Khalid M Alshamrani, Abdulkader A Alkenawi, Bushra N Alghamdi, Rawan H Honain, Haneen A Alshehri, Marwah O Alshatiri, Noor Mail, Ahmed Subahi, Shaza S Alsharif, Abdulaziz A Qurashi, Shrooq Aldahery, Reham Kaifi

**Affiliations:** 1 College of Applied Medical Sciences, King Saud bin Abdulaziz University for Health Sciences, Jeddah, SAU; 2 Research Office, King Abdullah International Medical Research Center, Jeddah, SAU; 3 Medical Imaging, Ministry of the National Guard - Health Affairs, Jeddah, SAU; 4 College of Science and Health Professions, King Saud bin Abdulaziz University for Health Sciences, Jeddah, SAU; 5 College of Applied Medical Sciences, Taibah University, Medina, SAU; 6 College of Applied Medical Sciences, University of Jeddah, Jeddah, SAU

**Keywords:** dose-area product, patient-based dose, common radiographic examination, digital radiology systems, international diagnostic reference level

## Abstract

This study aims to audit radiation doses of adult patients who underwent common diagnostic X-ray examinations and compare dose area product (DAP) values with the established International Diagnostic Reference Level (IDRLs). Retrospective cross-sectional records of 339-patients who underwent 699-radiographic examinations between October 2018 and March 2019 were obtained. Patient-related factors, exposure, and DAP data were recorded for the six most common examinations. The mean and 75th percentile of DAPs were recorded and compared to IDRLs values. The 75th percentiles of the locally measured DAPs were below IDRLs for all examinations except for lateral lumbar, AP, and lateral thoracic spine, in which DAP-75th-percentile exceeded all IDRLs by up to 40.7%, 2.8%, 365.5%, respectively. Considering the type of detector used, the mean of the locally measured DAPs significantly exceeded the UK DRLs for the lateral thoracic spine and lateral lumbar spine. Locally measured DAP values were below the IDRLs except for thoracic and lumbar spine projections, which significantly exceeded.

## Introduction

Plain radiography is a valuable and most cost-effective technique for diagnosis and screening purposes in medicine to begin a procedure with, constituting the largest contribution worldwide compared to all other diagnostic imaging examinations, and accounting for 53% of all imaging procedures reported in England between the year of 2018-2019 [1]. However, the major disadvantage is the radiation risks associated with the use of X-ray exposure. Thus, all the individual medical exposures should be justified and optimized in terms of imaging acquisition parameters, in order for the net benefits to greatly outweigh any potential risks of health side effects [2,3]. Radiation dose assessment is considered as an important aspect of dose management for patients undergoing radiographic examinations to determine the radiation exposure received and to improve the awareness of risks associated with small levels of ionizing radiation and the high dose differences reported among patients receiving the same form of radiography examinations [4]. To control the excessive dose to the patient, the Task Group 116 (American Association of Physicists in Medicine) identified a method of providing feedback, in the form of a standard index, to operators of digital radiographic systems, which reflects the adequacy of the exposure that has reached the detector after every exposure event [5]. It is of high priority and importance for the quality system of all types of radiographic imaging to reduce the radiation risk without compromising clinical efficiency [6] and to monitor the actual radiation dose in and across radiology departments utilizing suitable and well-defined procedures [7].

A guideline for optimal radiation dose must be followed to reduce the radiation risk to the patient. The International Commission on Radiological Protection (ICRP) has approved the use of the Diagnostic Reference Level (DRL) to protect patients from radiation risk [8,9]. DRL is defined as the absorbed dose in air, or tissue-equivalent material at the surface of a simple phantom or a representative patient, which was derived from a threshold (75th percentile) that is selected from the distribution of dose estimates obtained locally and collected regionally or nationally [10]. Both the ICRP [9] and the National Council on Radiation Protection and Measurements (NCRP) [11] recommend the use of the 75th percentile value as a national DRL. The appropriate use of the DRL method relies on the maximum possible uniformity of the patient size and the radiography procedure [12]. According to Charnock et al. (2013) [13], the DRL process is well-known to optimize radiation exposure for patients while maintaining image quality of diagnostic value, and to identify large doses that do not substantially relate to the clinical outcome of medical imaging examinations.

In surveys performed to acquire dose information for different procedures, it is important to identify radiation doses that are too low as well as too high, as both may have consequences for the patient [10]. Periodic comparison of local dose values to international DRL may uncover unusual dose creep or overexposure conditions for common radiographic procedures and therefore, provide a practical framework for quality improvement by promoting the need for corrective actions to be implemented when good practice is not applied, and for radiation protection optimization [10,14]. As a radiation dose descriptor, the dose area product (DAP) is a multiplication product between the patient exposed surface skin area in square centimeters or square meters and the surface radiation dose received in Grays [9,10]. The new digital radiographic machines allow the direct measurement of DAP values via the incorporation of a specific ionizing chamber mounted on the X-ray tube collimator surface [15].

Dosimetric DAP values auditing may help to achieve the optimization pillar of radiation protection (i.e., the procedure is performed at an optimized level with respect to the amount of radiation used) by ensuring the compliance of local health facilities with established national or international DRL for a standard diagnostic procedure (i.e., radiography), standard-sized patients, and for broadly defined types of equipment such as digital radiographic machines [8-10].

A detailed study to retrospectively review the DAP readings and technical parameters range for the plain radiography here in a local tertiary hospital are still missing or not done yet. Therefore, this work has been carried out to retrospectively review and audit the DAP readings for adult patients who underwent common diagnostic X-ray examinations performed using two digital radiographic units of a single hospital. This study further reports a comparison between the locally audited DAPs and the established international DRL.

## Materials and methods

Patient and examinations

A retrospective dose survey was conducted between October 2018 and March 2019; during which 5,634 diagnostic radiographic examinations encompassing six common X-ray procedures and a total of 10 individual projections were performed. Of the 5,634 examinations, a representative sample of 699 examinations for 339 adult patients (151 male, 188 female) were selected through non-probability convenience sampling and based on infinite/required sample size estimation technique described in [16]. The X-ray examinations/projections included were chest posterior-anterior (PA), chest lateral, cervical spine anterior-posterior (AP), cervical spine lateral, Thoracic spine AP, lumbar spine AP, lumbar spine lateral, pelvis AP, abdomen AP.

Inclusion criteria included protocoled adult examinations as defined in the Digital Imaging and Communications in Medicine (DICOM) header via the radiology information system (RIS) (Centricity RIS-i 6).

For each patient, demographic (age, gender), and anatomical (weight, height, body mass index (BMI)) data were obtained from the hospital information system (HIS) (Bestcare 2.0 A). For each examination, exposure [tube voltage (kVp), mAs factor (Milliamperage (mA)-Exposure time (s)], geometrical [source to image distance (SID)], and acquisition mode [manual, automatic exposure control (AEC)] were exported from the DICOM structure report. The dosimetric [dose area product (DAP)] data was obtained from the electronically collimated and cropped displayed radiograph that is used for review by radiologists.

Radiography equipment

The study was conducted at a single educational and tertiary hospital. The examinations were performed in two X-ray rooms, with Philips SMI DigitalDiagnost 2.0.x Dual Detector TH/VS digital radiography (DR) systems (Philips Medical Systems, Hamburg, Germany). Both systems are equipped with X-ray Tube housing assembly encompassing Permanent Filtration: 2.5 mm Al at 75 kVp, Nominal Voltage: 150 kV. No added filtration was utilized for the selected examinations.

To ensure the consistency of the equipment performance, the reliability and reproducibility of the exposure and the dosimetric parameters; the data was obtained immediately after the completion of the annual quality control (QC) testing for both machines. The two radiographic machines have passed the annual QC survey, which included visual inspections, and tests for generator, collimation, table/wall Bucky, resolution, uniformity, artifacts, ghost image, aliasing/grid, AEC circuit, and DAP verification test.

Ethical consideration

This study was conducted after obtaining an institutional review board (IRB) approval from the local authority, and data were collected after obtaining a permission from the radiology department head. Anonymity and confidentiality were maintained via linking each selected examination to an assigning reference number and keeping the collected data in a secure and encrypted Excel file.

Statistical analysis

Initial descriptive statistics (means (µ), standard deviations (σ), ranges, percentages) were generated to examine demographics, technical parameters, and DAP values variations among projections. The 75th-percentile of locally recorded DAPs were calculated and compared to IDRLs values range, which was acquired from digital and computed radiographic systems of five different countries [12,17-22]. The percentage 75th-percentile DAP differences were defined as:

$\delta _{75th percintile}= 100 \left (\frac{75 th percintile_{(Local DAPs)}- Maximun 75th percintile _{(IDRL DAPs)}}{Maximum 75 th percintile _{_{(IDRL DAPs)}}} \right )$

was used to describe DAP changes between the local measurement and the maximum values of IDRLs range.

For each projection, correlation between BMI, exposure parameters and DAP values was performed by first, examining data distribution normality using Shapiro-Wilk test and second, Pearson's correlation test is used to examine the association between the continuous variables.

Using one sample t-test and general linear model univariate analysis with BMI as a covariate; a focused-analysis considering comparisons among digital radiographic systems only was performed to examine the difference between the locally measured DAP means and the 2012-published UK DRL data [21], in which the DAP mean for lateral chest was not indicated. Statistical tests were conducted at a significance level of 0.05.

## Results

Patients' characteristics and technical parameters for the selected examinations are shown in Table 1. Of a sample of 339-adult patients and a total of 699-radiographic examinations of 10 projections; 138 (19.7%) were chest x-rays (PA 71; lateral 67), 137 (19.6%) were cervical spine x-rays (AP 68; lateral 69), 50 (7.2%) were thoracic spine X-rays (AP 25; lateral 25), 287 (41.1%) were lumbar spine X-rays (AP 144; lateral 143), 32 (4.6%) were AP abdominal X-rays, and 55 (7.9%) were AP pelvis X-rays.

**Table 1:** Demographics, and key technical parameters. PA: posterior-anterior; AP: anterior-posterior; SID: source to image distance.

Examination	Projection (number)	Patient characteristics		Technical parameters		
		Age mean (year)/SD	BMI mean (kg/m^2^)/SD	kVp mean/(range)	mAs mean/(range)	SID mean (cm)/(range)
Chest	PA (n = 71)	44.9/19.3	28.4/7.45	125.00	1.5/(0.40-3.70)	174.48/(149.90-179.50)
	Lateral (n = 67)	45.2/19.5	28.8/7.4	125.00	5.29/(0.25-19.70)	176.3/9.7 (118.8-183.2)
Cervical spine	AP (n = 68)	50.7/13.7	30.6/6	70.22/(70.00-85.00)	8.12/(1.90-25.00)	152.51/(99.90-185.20)
	Lateral (n = 69)	50.2/14.4	30.45/6	77.72/(77.00-85.00)	6.23/(1.00-24.80)	153.44/(96.70-179.50)
Thoracic spine	AP (n = 25)	52.80/18.8	27.8/9.8	77.16/(77.00-81.00)	17.16/(3.20-62.90)	116.72/(100.00-158.50)
	Lateral (n = 25)	50.92/20	28.03/10.3	72.08/(66.00-85.00)	114.40/(9.90-249.90)	121.69/(99.90-178.10)
Lumbar Spine	AP (n = 144)	52.35/15.4	30.5/6.3	77.78/(77.00-125.00)	49.74/(2.20-308.80)	128.80/(96.70-179.30)
	Lateral (n = 144)	52.35/15.4	30.5/6.3	85.34/(66.00-91.00)	85.85/(5.00-249.80)	129.65/(96.70-179.30)
Abdomen	AP (n = 32)	48.84/17.1	27.42/4.5	77.00	20.18/(4.60-66.00)	120.00/(100.00-147.30)
Pelvis	AP (n = 55)	43.73/22.7	27.32/7.5	79.89/(73.00-81.00)	21.92/(2.60-268.50)	120.85/(92.50-178.10)

The subjects’ age range in years per examination and for all projections are 12-89 for chest, 13-83 for cervical spine, 12-83 for thoracic spine, 17-86 for lumbar spine, 18-79 for abdomen, and 12-87 for pelvis X-rays. The subjects’ BMI range (kg/m^2^) per examination and for all projections are 14.03-55.5 for chest, 18.6-47.8 for cervical spine, 18.6-52 for thoracic spine, 16.6-48 for lumbar spine, 17.1-37.3 for abdomen, and 14.4-46.4 for pelvis X-rays. 

The kVps for the selected examinations and for all projections are 125 kV for chest, 70-85 kV for cervical spine, 66-85 kV for thoracic spine, 66-125 kV for lumbar spine, 77 kV for abdomen, and 73-81 for pelvis X-rays. The mAs range for the selected examinations and for all projections are 0.25-19.7 for chest, 1-25 for cervical spine 3.2-249.9 for thoracic spine, 2.2-308.8 for lumbar spine, 4.6-66 for abdomen, and 2.6-268.5 for pelvis X-rays. The SID range (cm) for the selected examinations and for all projections are 118.8-183.2 for chest, 96.7-185.2 for cervical spine, 99.90-178.10 for thoracic spine, 96.7-179.3 for lumbar spine, 100-147.3 for abdomen, and 92.5-178.1 for pelvis X-rays. 

**Figure 1:**
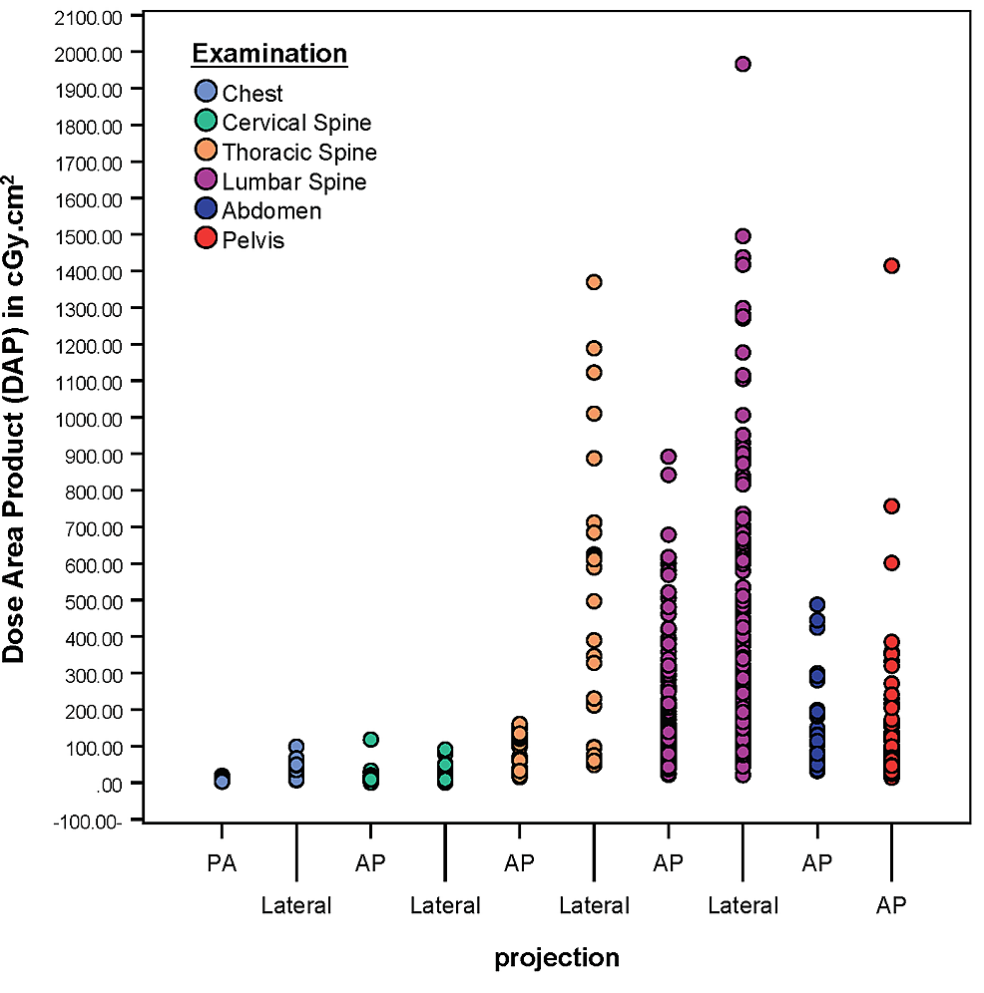
DAPs variations among examinations/projections. PA: posterior-anterior; AP: anterior-posterior; DAP: dose area product.

The distribution of DAP values for the selected examinations and per projection are illustrated in Figure 1. The supporting numerical data (µ and σ) is listed in Table 4. Compared to chest and cervical spine x-rays, a wider dispersion and increased variability was observed and evidenced by a larger standard deviation for thoracic spine (lateral σ = 391.55), lumbar spine (AP σ = 160.82; lateral σ = 368.34), abdomen (σ = 123.93), and pelvis (σ = 223.76).

Pearson's correlation results are listed in Table 2. First, BMI demonstrated a statistically significant and positive correlation with DAP values for all X-ray examinations regardless of the projection (P < 0.0001 for chest PA and lateral; P = 0.004 for cervical spine AP and P = 0.02 for cervical spine lateral; P < 0.0001 for thoracic spine AP and P = 0.005 for thoracic spine lateral; P < 0.0001 for lumbar spine, abdomen, and pelvic examinations). Second, mAs demonstrated a statistically significant and positive correlation with DAP values for all X-ray examinations regardless of the projection (P < 0.0001 for all examinations). Third, kVp demonstrated: (a) statistically significant and positive correlation with DAP values for lateral cervical and thoracic X-ray examinations (P < 0.0001; P = 0.009, respectively), and (b) statistically significant and negative correlation with DAP values for lateral lumbar X-ray examinations (P < 0.0001). Fourth, there is no correlation between SID and DAP values of all examinations.

**Table 2:** Correlation of DAPs vs anatomical (BMI), exposure (mAs, kVp), and geometrical (SID) parameters. PA: posterior-anterior; AP: anterior-posterior; DAP: dose area product; SID: source to image distance.

	Examination	Projection (number)	Pearson correlation	BMI	mAs	kVp	SID
DAP	Chest	PA (71)	Coefficient r	0.68	0.91	-	-0.15
			P-value	<0.0001	<0.0001	-	0.2
		Lateral (67)	Coefficient r	0.73	0.88	-	0.19
			P-value	<0.0001	<0.0001	-	0.1
	Cervical spine	AP (68)	Coefficient r	0.35	0.77	0.15	0.05
			P-value	0.004	<0.0001	0.2	0.7
		Lateral (69)	Coefficient r	0.29	0.86	0.51	0.17
			P-value	0.02	<0.0001	<0.0001	0.19
	Thoracic spine	AP (25)	Coefficient r	0.88	0.95	0.15	0.29
			P-value	<0.0001	<0.0001	0.49	0.17
		Lateral (25)	Coefficient r	0.56	0.94	0.51	0.22
			P-value	0.005	<0.0001	0.009	0.33
	Lumbar spine	AP (144)	Coefficient r	0.70	0.84	0.11	0.1
			P-value	<0.0001	<0.0001	0.2	0.26
		Lateral (143)	Coefficient r	0.36	0.85	-0.37	-0.08
			P-value	<0.0001	<0.0001	<0.0001	0.44
	Abdomen	AP (32)	Coefficient r	0.75	0.94	-	-0.16
			P-value	<0.0001	<0.0001	-	0.38
	Pelvis	AP (55)	Coefficient r	0.76	0.93	0.13	-0.11
			P-value	<0.0001	<0.0001	0.34	0.4

**Figure 2:**
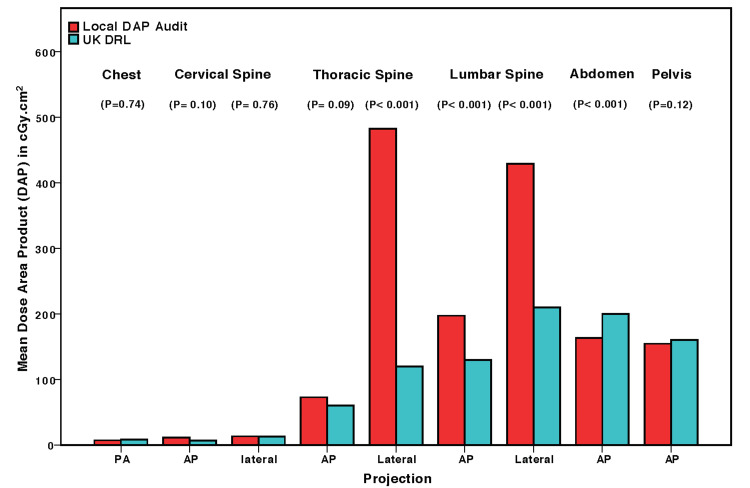
Focused-analysis of flat panel-based digital radiographic systems only: comparisons of local DAPs vs UK DRL (adjusted for BMI). PA: posterior-anterior; AP: anterior-posterior; DAP: dose area product; DRL: Diagnostic Reference Level.

The main results of DAPs 75th percentiles are listed in Table 3. First, the 75th percentiles of the locally measured DAPs were below IDRLs 75th percentiles for chest, cervical spine, lumbar spine (AP), abdomen, and pelvis examinations. Second, the 75th percentiles of the locally measured DAPs exceeded the maximum range of IDRLs 75th percentiles for thoracic spine [AP (2.8%), lateral (365.5%)], and lateral lumber spine (40%) X-rays. When the type of detector is considered, and without statistically adjusting for BMI, the mean of the locally measured DAPs significantly exceeded the UK DRLs for the following examinations: Lateral thoracic spine (P < 0.0001), AP and lateral lumbar spine (P < 0.0001), as listed in (Table 4). This significant increase still exists even after statistically controlling for BMI [lateral thoracic (P < 0.0001), AP and lateral lumbar spine (P < 0.0001), as listed in Table 4, and illustrated in Figure 2.

**Table 3:** Comparison of the local DAP audit vs IDRLs. DAP: dose area product; IDRL: International Diagnostic Reference Level; PA: posterior-anterior; AP: anterior-posterior.

Examination	Projection (number)	Local DAP audit		IDRLs	δ_75th Percentile _ (%)
		75^th ^percentile (cGy.cm^2^)	95% confidence interval for the 75^th^ percentile	75^th^ percentile (range) (cGy.cm^2^)	
Chest	PA (n = 71)	8.84	(7.32-10.93)	(10-30)	-70.5
	Lateral (n = 67)	32.48	(28.18-39.89)	60	-45.9
Cervical spine	AP (n = 68)	12.27	(10.30-17.18)	(15-40)	-69.3
	Lateral (n = 69)	11.04	(9.07-15.77)	(15-40)	-72.4
Thoracic spine	AP (n = 25)	123.34	(71.54-146.11)	(100-120)	2.8
	Lateral (n = 25)	698.25	(603.35-1066.20)	(140-150)	365.5
Lumbar spine	AP (n = 144)	253.78	(212.54-305.70)	(15-300)	-15.4
	Lateral (n = 144)	633.09	(491.31-722.16)	(250-450)	40.7
Abdomen	AP (n = 32)	197.85	(143.90-297.82)	(250-275)	-28.1
Pelvis	AP (n = 55)	172.79	(128.70-318.58)	(220-350)	-50.6
= Increase IDRLs = International Dose Reference Levels of 5 countries δ_75th Percentile_=100 75th Percentile_Local DAPs_ - Maximum 75th Percentile_ IDRL DAPs_Maximum 75th percentile_IDRL DAPs_					

**Table 4:** Focused-analysis of flat panel-based digital radiographic systems only: comparisons of local DAPs vs UK DRL. PA: posterior-anterior; AP: anterior-posterior; DAP: dose area product; DRL: Diagnostic Reference Level.

Examination	Projection (number)	Local DAP audit						UK DRL
		Before controlling for BMI			After controlling for BMI			
		Mean (cGy.cm^2^)	Standard deviation	P-value	Mean (cGy.cm^2^)	Standard deviation	P-value	Mean (cGy.cm^2^)
Chest	PA (71)	7.3	3.22	0.075	7.09	23.28	0.74	8
	Lateral (67)	25.54	17.12	-	25.59	23.38	-	-
Cervical spine	AP (68)	11.61	14.81	0.013	11.54	22.49	0.1	7
	Lateral (69)	13.11	17.42	0.96	13.22	22.33	0.76	13
Thoracic spine	AP (25)	72.83	50.43	0.22	72.83	36.83	0.09	60
	Lateral (25)	501.21	391.55	<0.0001	482.49	37.19	<0.0001	120
Lumbar spine	AP (144)	194.22	160.82	<0.0001	197.38	15.53	<0.0001	130
	Lateral (143)	434.79	368.34	<0.0001	428.93	15.59	<0.0001	210
Abdomen	AP (32)	158.81	123.94	0.07	163.16	33.05	<0.0001	200
Pelvis	AP (55)	162.23	223.77	0.95	154.54	26.02	0.13	160

## Discussion

The primary findings of this study are that compared to chest, cervical spine, abdomen, and pelvis X-rays; lateral thoracic and lumbar spine examinations show: (a) wider dispersion and increased DAP values variability, (b) an increase in DAPs 75th percentile by 365.5% for lateral thoracic and 40% for lateral lumber X-rays, relative to the maximum range of IDRLs 75th percentiles, (c) an increase in mean DAP values (before and after adjusting for BMI), relative to DAPs UK DRLs of digital radiographic detectors/systems only.

A wide variation of the tube load (mAs) selected was observed for different patients’ BMI and for thoracic spine (lateral), lumbar spine (AP, lateral) and pelvis examinations in particular. Such variation in addition to the positive correlation between mAs and DAPs may explain the increased DAPs variability seen in Figure 1 for these examinations (σ = 391.55; 160.82; 368.34; 223.76, respectively). Compared to IDRLs and as listed in Table 3 and Table 4, the marked increase in the DAP 75th percentile and mean observed in lateral thoracic spine and lumbar spine examinations could be attributed to the selection of high mAs technique and wide-field size. This significant increase still exists even after statistically controlling for BMI, and also considering the type of radiographic system (i.e., flat-panel based digital radiographic systems), which is reflective of either poor calibration regarding the programmed reference exposure technique via AEC system or suboptimal technologists’ practices with regards to the choice of radiographic technique (i.e., mAs) or oversized collimation.

The high mAs technique seen for lateral lumbar and thoracic examination could be due to inaccuracy in the manufactures’ defaulted and programmed reference exposure technique or inaccurate manual adjustment of exposure factors by the radiology technologist in response to variations in patient size that exceeded the recommended exposure technique provided by the manufacturers, which can lead to an increase in the incident dose and therefore a dose creep or overexposure that can easily go unnoticed [23-26].

In this study and for all radiographic examinations included, the actual field size was not retrievable owing to the unavailability of the field collimation details in the DICOM header of each image and the possible improper electronic collimation, which may have hidden the actual field dimension of the irradiated body part [24]. Possible systematic patient overexposure due to poor collimation practices may have been concealed with the use of the electronically collimated radiograph for diagnosis [24,27-30]. However, it cannot be determined whether the marked increase in the DAP values observed in lateral thoracic spine and lumbar spine examinations is attributable to high dose value or a large field size or both, as field size information is unavailable. 

The limitations of this study include (a) the image quality was not evaluated to better apply the as low as reasonably achievable (ALARA) principle; so, future studies need to be conducted in which DAP and image quality are both investigated in order to develop and maintain a proper radiation protection culture in diagnostic radiography, (b) since the field collimation data are unrecorded in the DICOM header and/or concealed by the cropped image, it is difficult to differentiate whether the DAPs differences relative to IDRLs are due to high dose value secondary to improper technical factor selection or oversized collimation, or both; therefore, reconfiguration of the DICOM structure report are recommended in which the field size dimensions are automatically registered and stored. Moreover, radiologists are encouraged to use the original radiograph for review and interpretation in order to detect any occasional overexposure of patients secondary to poor collimation practices, as using electronically cropped radiographs for review may hide a possible oversized collimation, which can lead to a systematic patients’ overexposure that can go unnoticed [24,29].

In this study, the retrospective dose audit is carried out to evaluate the current dose details on common radiographic examinations. The mean and 75th percentile DAP data reported herein are obtained from two digital X-ray units of a single hospital. Such data could be used as a preliminary effort to initiate the establishment of regional DRLs (if not existed) and as a reference for future local DAPs audit comparison. Although the image quality is not evaluated in this study; the wide DAPs variation recorded particularly for lateral thoracic and lumber X-ray examinations is an indication that considerable dosage decrease may well be achieved while maintaining an acceptable image quality [31]. Thus, the radiology department and the imaging staff are encouraged to make the necessary effort to optimise their examination protocols and collimation practices in order to avoid unnecessary exposure to patients by maintaining the dose as low as reasonably achievable, while keeping the image quality at an acceptable level.

## Conclusions

Locally measured DAP values were below the IDRLs except for thoracic and lumbar spine projections, which exceeded. Future dosimetric reassessment is recommended after (a) optimizing the protocol of these projections to ensure that DAP values are back within the IDRLs range, (b) calibrating the manufactures’ AEC system to optimize the programmed reference exposure technique for thoracic and lumbar spine examinations, (c) reconfiguration of DICOM structure report to automatically record the actual radiation field size, and (d) ensuring optimal collimation practices via increasing the awareness towards the systematic overexposure risk associated with electronic collimation, and the need to use the original images for review and interpretation, which can help uncovering the occasional oversized collimation errors that can be concealed when the masked or cropped images are solely used for interpretation. This study may serve as a baseline reference for future research in which both dose and image quality are examined for optimization purposes, to ensure patient safety, and to develop and maintain an optimal radiation protection culture in diagnostic radiography.

## References

[1] Dixon S (2019). Dixon S. Diagnostic Imaging Dataset Statistical Release. NHS England and NHS Improvement. https://www.england.nhs.uk/statistics/wp-content/uploads/sites/2/2019/07/Provisional-Monthly-Diagnostic-Imaging-Dataset-Statistics-2019-07-18.pdf.

[2] UNION TCOTE (2014). UNION TCOTE: COUNCIL DIRECTIVE 2013/59/EURATOM of 5 December 2013 laying down basic safety standards for protection against the dangers arising from exposure to ionising radiation, and repealing Directives 89/618/Euratom, 90/641/Euratom, 96/29/Euratom, 97/43/Euratom and 2003/122/Euratom. Official Journal of the European Union. Directives 89/618/Euratom, 90/641/Euratom, 96/29/Euratom.

[3] Hall EJ, Brenner DJ (2008). Cancer risks from diagnostic radiology. Br J Radiol.

[4] Huda W, Nickoloff EL, Boone JM (2008). Overview of patient dosimetry in diagnostic radiology in the USA for the past 50 years. Med Phys.

[5] Shepard SJ, Wang J, Flynn M (2009). An exposure indicator for digital radiography: AAPM Task Group 116 (executive summary). Med Phys.

[6] Spelic DC, Kaczmarek RV, Suleiman OH (2004). Nationwide Evaluation of X-ray Trends survey of abdomen and lumbosacral spine radiography. Radiology.

[7] Baiter S, Rosenstein M, Miller DL, Schueler B, Spelic D (2011). Patient radiation dose audits for fluoroscopically guided interventional procedures. Med Phys.

[8] ICRP P 105 (2007). Radiation protection in medicine. Ann ICRP.

[9] (2007). The 2007 Recommendations of the International Commission on Radiological Protection. ICRP publication 103. Ann ICRP.

[10] Vañó E, Miller DL, Martin CJ (2017). ICRP Publication 135: Diagnostic Reference Levels in Medical Imaging. Ann ICRP.

[11] (2012). NCRP RNo 172: Reference Levels and Achievable Doses in Medical and Dental Imaging: Recommendations for the United States. National Council on Radiation Protection and Measurements. https://ncrponline.org/publications/reports/ncrp-report-172/.

[12] Hart D, Hillier MC, Wall BF (2009). National reference doses for common radiographic, fluoroscopic and dental X-ray examinations in the UK. Br J Radiol.

[13] Charnock P, Moores BM, Wilde R (2013). Establishing local and regional DRLs by means of electronic radiographical X-ray examination records. Radiat Prot Dosimetry.

[14] Teunen D (1998). The European Directive on health protection of individuals against the dangers of ionising radiation in relation to medical exposures (97/43/EURATOM). J Radiol Prot.

[15] Nickoloff EL, Lu ZF, Dutta AK, So JC (2008). Radiation dose descriptors: BERT, COD, DAP, and other strange creatures. Radiographics.

[16] Cochran WG (1965). Sampling techniques. Biometrics.

[17] Roch P, Célier D, Dessaud C, Etard C (2018). Using diagnostic reference levels to evaluate the improvement of patient dose optimisation and the influence of recent technologies in radiography and computed tomography. Eur J Radiol.

[18] Veit R, Guggenberger R, Nosske D, Brix G (2010). Diagnostic reference levels for X-ray examinations: update 2010. Radiologe.

[19] Vanaudenhove T, Slambrouck KV, Fremout A Niveaux de référence diagnostiques nationaux en radiologie - Deuxième itération pour les examens de radiologie conventionnelle, mammographie et radiologie interventionnelle. https://afcn.fgov.be/fr/system/files/rapport-rx-iteration-2-ct-iteration-4.pdf.

[20] Notice R (2011). Notice R-06-04: Niveaux de référence diagnostiques (NRD) en radiologie par  projection. Office fédéral de la santé publique (OFSP). https://www.astrm.ch/files/Dokumente/Verband/Fachstellen/Strahlenschutz/R-06-04_niveaux_de_reference_diagnostiques__NRD__en_radiologie_par_projection.pdf.

[21] Hart D, Hillier MC, Shrimpton PC (2012). Doses to patients from radiographic and fluoroscopic X-ray imaging procedures in the UK - 2010 review. Health Protection Agency Centre for Radiation, Chemical and Environmental Hazards.

[22] Strahlenschutz B für (2016). Strahlenschutz B für: Bekanntmachung  der aktualisierten diagnostischen Referenzwerte  für diagnostische und interventionelle Röntgenanwendungen. Published Online First: 22 June.

[23] Ching W, Robinson J, McEntee M (2014). Patient-based radiographic exposure factor selection: a systematic review. J Med Radiat Sci.

[24] Tsalafoutas IA (2018). Electronic collimation of radiographic images: does it comprise an overexposure risk?. Br J Radiol.

[25] Shi C (2005). Specifications, performance evaluations, and quality assurance of radiographic and fluoroscopic systems in the digital era. Med Phys.

[26] Shah C, Jones AK, Willis CE (2008). Consequences of modern anthropometric dimensions for radiographic techniques and patient radiation exposures. Med Phys.

[27] Guðjónsdóttir J (2019). The unnecessary dose behind cropped radiographs. Radiography Open.

[28] Pazanin A, Skrk D, O'Driscoll JC, McEntee MF, Mekis N (2020). Optimal collimation significantly improves lumbar spine radiography. Radiat Prot Dosimetry.

[29] Bomer J, Wiersma-Deijl L, Holscher HC (2013). Electronic collimation and radiation protection in paediatric digital radiography: revival of the silver lining. Insights Imaging.

[30] Zetterberg LG, Espeland A (2011). Lumbar spine radiography--poor collimation practices after implementation of digital technology. Br J Radiol.

[31] Jibiri NN, Olowookere CJ (2016). Evaluation of dose-area product of common radiographic examinations towards establishing a preliminary diagnostic reference levels (PDRLs) in Southwestern Nigeria. J Appl Clin Med Phys.

